# Risk factors for endotracheal re-intubation following coronary artery bypass grafting

**DOI:** 10.1186/1749-8090-8-208

**Published:** 2013-11-09

**Authors:** Liu Jian, Shi Sheng, Yu Min, Yuan Zhongxiang

**Affiliations:** 1 Cardiovascular surgery department, Shanghai First People’s Hospital, Shanghai Jiaotong University, 100 Haining Rd, Hongkou District, Shanghai 200080, China

**Keywords:** Coronary artery bypass grafting, Risk factors, Complication

## Abstract

**Background:**

Endotracheal re-intubation following coronary artery bypass grafting (CABG) is often associated with significant morbidity and mortality. However, few reports have focused on the independent risk factors for re-intubation following CABG. This study aimed to evaluate the independent risk factors for re-intubation following CABG.

**Methods:**

The pre-, intra-, and post-operative materials in patients who had selective and isolated CABG performed on them from January 2004 to July 2012 in our hospital were analyzed retrospectively. Unvariate analysis and logistic regression were used to analyze the risk factor of postoperative re-intubation following CABG.

**Results:**

Among the 1,244 patients investigated, 97 cases suffered from postoperative re-intubation, and the incidence rate of postoperative re-intubation was 7.8%. The in-hospital mortality in the re-intubation group was significantly higher than that in the non-re-intubation group (9.3% versus 1.4%, P = 0.004). Re-intubation also correlated with many negative outcomes such as pneumonia, tracheotomy, acute renal failure, infection of incision, prolonged mechanical ventilation time, prolonged intensive care unit (ICU) stay and prolonged hospital stay. The most commonly cause of re-intubation after CABG was hypoxemia due to cardiogenic and noncardiogenic disease, which accounted for 72.2%. The relative factors of postoperative re-intubation were tested through unvariate analysis and logistic regression, and the associated factors were obtained. The associated factors for re-intubation following CABG included preoperative chronic obstructive pulmonary disease (COPD) (OR = 2.134, 95% CI = 1.472-2.967), preoperative congestive heart failure (CHF) (OR = 2.325, 95% CI = 1.512-3.121), postoperative relative hypoxemia (OR = 2.743, 95% CI = 1.657-3.326), postoperative acute kidney injury (AKI) (OR = 2.976, 95% CI = 2.127-4.023), postoperative total mechanical ventilation time (OR = 1.976, 95% CI = 1.347-2.645).

**Conclusion:**

Preoperative COPD, preoperative CHF, postoperative relative hypoxemia, postoperative AKI and postoperative total mechanical ventilation time were five independent risk factors for re-intubation following CABG.

## Background

With the aging of the population, rising incidence of coronary artery disease (CAD) and surgical improvement of CAD, more and more elderly patients have to be performed with coronary artery bypass grafting (CABG), and the pulmonary complications seem to be on the increase. Some patients need to be re-intubated after the first extubation. Some reports revealed that many factors and the interaction of these factors leads to postoperative pulmonary complications following CABG [1,2]. In this study, the pre-, intro- and post- operative materials in patients undergoing selective and isolated CABG from January 2004 to July 2012 were collected and analyzed retrospectively. The associated factors were tested through descriptive analysis and logistic regression, the associated factors of postoperative re-intubation were obtained, and the aim is to prevent and processing the conditions that led to re-intubation and avoid the condition of ischemia and hypoxia between the extubation and re-intubation after CABG.

## Methods

### Patients

The study was approved by the institutional medical ethics committee and was consistent with the spirit of the Declaration of Helsinki. Institutional Review Board of Shanghai First People’s Hospital affiliated to Shanghai Jiaotong University reviews all research. From January 2004 to July 2012, 1637 patients suffering from CAD underwent CABG. Among them 295 patients underwent CABG combined with valve surgery and were excluded. The remaining 1342 patients underwent isolated CABG. We also excluded the patients extubated after 48 hours or re-intubated because of non-breathing problems such as heart arrest, re-operation for bleeding. Thus, 1,244 consecutive patients (912 males and 332 females, with a mean age of 67.4 ± 9.7 years) were brought into the study. Among them 97 cases were re-intubated. Coronary artery angiography revealed double vessel disease in 209 patients and triple vessel disease in 1035 patients, and 260 cases were concurrent with left main trunk disease; 32 cases were concurrent with left ventricular aneurysm. The 344 cases were performed with off-pump CABG and 900 cases with an on-pump CABG. The number of bypass graftings ranged from 1 to 5 ( mean 2.98 per case). Left internal mammary artery was used as a bypass conduit in 1189 (95.6%) cases and great saphenous vein graft in 1135 (91.2%) cases. The operation was performed in Inhalation-Intravenous General Anesthesia. Anesthesia was induced with midazolam (2-3 mg), fentanyl (0.2 mg), propofol (0.5-1.5 mg/kg) and vecuronium and maintained with isoflurane and continuous infusion of propofol (2 to 5 mg/kg/h); 0.1-0.2 mg fentanyl was intravenously administered before skin incision, sternotomy, aortic cannulation and initiation of cardiopulmonary bypass, respectively; total amount of fentanyl was less than 15 μg/kg during operation). Mid-sternal incision was performed in all cases. A blood gas analyzer (i-STAT Corporation, East Windsor, NJ,USA) was used to measure the arterial partial pressure of oxygeon (PaO_2_) and carbon dioxide (PaCO_2_) peri-operatively. All post-operative patients were ventilated by Drager Savina respirator (Drägerwerk AG & Co. KGaA, Lübeck, Germany).

### Criteria for extubation and re-intubation

Criteria for extubation included an alert and hemodynamically stable patient with no excessive bleeding, ability of the patient to breathe with simultaneous intermittent mandatory ventilation (machinery rate 4 breaths/min ,no pressure support) for at least 30 minutes with a fraction of inspired oxygen of less than 0.60 and a total respiratory rate less than 25 breaths/min, an arterial blood PO_2_ greater than 70 mmHg, a PaCO_2_ less than 50 mmHg and a pH greater than 7.35, with no metabolic acidosis. Other criteria included a mandatory chest radiograph before extubation to rule out pneumothorax, pleural effusion and atelectasis.

Criteria for re-intubation included the condition with severe dyspnea and respiratory rate more than 35 breaths/min, apparent acceleration of heartbeat and elevation of blood pressure compared to the condition of extubation, an arterial blood PO_2_ less than 60 mmHg or a PaCO_2_ greater than 50 mmHg, a repeated pH lesser than 7.35 with or without respiratory acidosis, a large-area pneumonia or atelectasis.

### Investigated data

From January 2004 to July 2012, the charts of all patients received isolated CABG in our hospital were reviewed. The relevant pre-, intro- and post- operative data of all selected cases were investigated and retrospectively analyzed. The pre-operative materials were as follows: sex, age, body mass index (BMI), smoking history and smoking index, preoperative acute cardiac infarction (AMI) (evidence of AMI within the last 30 days before surgery), preoperative renal dysfunction (creatinine more than 2.0 mg/dl or requiring dialysis), preoperative hypertension, preoperative diabetes, preoperative severe chronic obstructive pulmonary diseases (COPD) ( FEV_1_/FVC ratio < 70%, FEV_1_ < 50% predicted), pre-operative left ventricular ejection fractions (LVEF) and left ventricular end-diastolic diameter (LVEDD), preoperative congestive heart failure (CHF, New York Heart Association (NYHA) class III and IV), preoperative PaO_2_ and PaCO_2_ (the value of preoperative PaO_2_ and PaCO_2_ were obtained under no oxygen supply), hypoalbuminemia (serum albumin less than 30 g/L), preoperative left ventricular aneurysm, left main trunk disease, the number of diseased vessels. Intraoperative data included CABG with or without cardiopulmonary bypass, the duration of cardiopulmonary bypass and aortic cross-clamp, bypass graftings, the duration of the operation. Postoperative information included relative hypoxemia (the last PaO_2_ before extubation between 70 mmHg and 90 mmHg), relative hypercapnia (the last PaCO_2_ before extubation between 45 mmHg and 50 mmHg), pneumonia (Pulmonary inflammatory exudation in chest X-ray and positive sputum culture), low cardiac output syndrome (central venous pressure > 18 cmH_2_O for at least 2 hours, cardiac index < 2.5 L/m^2^, relative decreasing > 20% in the arterial systolic pressure compared with basic line for at least 2 hours, difference of central and peripheral body temperature > 5°C, conforming to the above two) , perioperative AMI (new Q-wave infarction within 48 h after surgery), requirement of intra-aortic balloon pump (IABP), atrial fibrillation (AF), ventricular fibrillation (VF), acute kidney injury (AKI) (absolute increase >0.3 mg/dl or relative increase >50% in the serum creatinine level compared to the preoperative baseline value), stroke (new permanent neurological event with evidence of computed tomography, such as cerebral infarction or hemorrhage), bleeding requiring re-operation (re-operation to control bleeding within 36 h hours following initial surgery), infection of incision, total mechanical ventilation time, tracheotomy, length of intensive care unit (ICU) stay, length of hospital stay and hospital mortality (death during same admission or within 30 days after surgery upon discharge).

### Statistically analysis

Statistical analysis was performed using the SPSS17.0 statistical software package. All *p* values <0.05 were considered to be statistically significant. Each of the peri-operative materials were defined as independent variables; post-operative re-intubation (or not) following extubation was defined as a dependent variable. Univariate analysis, using the unpaired t-test or Satterthwaite's approximate t test according to homogeneity test for variance to compare measurement data and chi-square or Fisher's exact test to compare enumeration data, was performed to assess statistically significant variables, and those with *p* < 0.10 were then entered into a stepwise logistic regression analysis to identify the independent risk factors for re-intubation. The Hosmer-Lemeshow goodness of fit coefficient was computed for the regression model.

## Results

1,244 consecutive patients who underwent isolated CABG and were extubated within 48 hours were entered into this study. Among them, 97 cases were re-intubated because of breathing problems, accounting for 7.8%. The mean intubation time in this group was 118.4 ± 87.3, while the other group 17.4 ± 5.5 hours.

Among the re-intubation group, tracheotomy occurred in 28 cases. Complications after re-intubation, the length of ICU and hospital stay and in-hospital mortality are shown in Table 1. There were significant difference in some morbidities and mortality such as AKI, prolonged mechanical ventilation time, length of ICU stay, length of hospital stay and hospital mortality. Patients in the re-intubation group had longer time of mechanical ventilation, length of ICU and hospital stay and higher hospital mortality.

**Table 1:** Comparison of morbidities and mortality between the two groups

	**Re-intubation**	**Non-re-intubation**	**P **
	**n = 97**	**n = 1147**	
Pneumonia	32(33%)	68(5.9%)	0.01
Tracheotomy	28(28.9%)	0(0%)	<0.001
LCOS	8(8.2%)	46(4%)	0.09
AMI	1(1%)	3(0.3%)	0.08
AF	10(10.3%)	87(7.6%)	0.43
VF	1(1%)	2(0.1%)	0.08
**AKI**	12(12.4%)	19(1.7%)	0
Stroke	2(2.1%)	25(2.2)	0.96
Infection of incision	5(5.1%)	18(1.6%)	0.04
TMVT(days)	5.2 ± 3.5	1.6 ± 0.5	0
ICU stay(days)	5.9 ± 3.6	2.7 ± 0.7	0.01
Hospital stay(days)	14.8 ± 5.3	8.5 ± 2.6	0.01
Hospital mortality	9(9.3%)	16(1.4%)	0

LCOS: low cardiac output syndrome; AMI: acute myocardial infarction; IABP: intro-aortic balloon pump; AF: atrial fibrillation; VF: ventricular fibrillation; AKI: acute kidney injury; TMVT: total mechanical ventilation time; ICU: intensive care unit.

**Figure 1:**
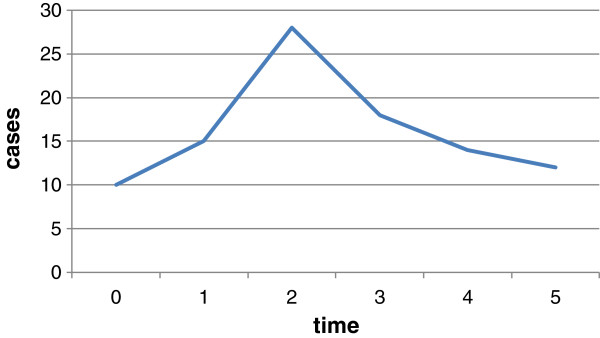
**Time points of re-intubation following extubation.** Time points of re-intubation following extubation. Time point 0: the same day as extubation. Time point 1: the first day after extubation. Time point 2: the second day after extubation. Time point 3: the third day after extubation. Time point 4: the fourth day after extubation. Time point 5: after fourth days following extubation.

**Figure 2:**
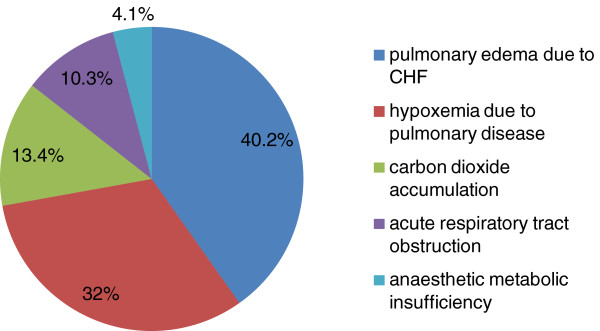
Reasons for re-intubation following CABG.

Re-intubation occurred mostly on the second day after extubation (Figure 1). The most commonly reason for re-intubation was pulmonary edema due to CHF. The others are as follows: hypoxemia due to pulmonary disease, carbon dioxide accumulation, acute respiratory tract obstruction, anaesthetic metabolic insufficiency (Figure 2). The four cases of re-intubation due to anaesthetic metabolic insufficiency all occurred in the same day of extubation.

Results of univariate analysis are shown in Table 2. It can be observed that age, BMI, preoperative COPD, preoperative CHF, preoperative LVEF, preoperative PaO_2_, postoperative hypoxemia, postoperative pneumonia, postoperative AKI and time of mechanical ventilation before re-intubation were relative risk factors for re-intubation following CABG.

**Table 2:** Comparison of pre-, intro- and post-operative data between the two groups

	**Re-intubation**	**Non-re-intubation**	**P **
	**n = 97**	**n = 1147**	
**Preoperation**			
Female	25(25.8%)	268(23.4%)	0.65
Age (years)	64.6 ± 7.2	62.5 ± 8.1	0.05
BMI (kg/m2)	25.8 ± 2.3	24.8 ± 1.8	0.04
Smoking history	36(37.1%)	325(28.3%)	0.38
AMI	15(15.5%)	67(5.8%)	0.09
Renal dysfunction	5(5.1%)	32(2.8%)	0.25
Hypertension	43(44.3%)	526(45.9%)	0.71
Diabetes	29(29.9%)	214(18.7%)	0.12
Severe COPD	26(26.8%)	52(4.5%)	0.01
LVEF	0.52 ± 0.11	0.55 ± 0.08	0.02
LVEDD	52.9 ± 7.9	51.3 ± 5.5	0.07
CHF	21(21.6%)	48(4.2%)	0.01
PaO2 (mmHg)	72.9 ± 11.5	68.3 ± 8.6	0.04
PaCO2 (mmHg)	41.3 ± 4.1	40.9 ± 3.6	0.09
Hypoalbuminemia	2(2.1%)	21(1.8%)	0.81
Ventricular aneurysm	3(3.1%)	25(2.2%)	0.54
Left main trunk disease	8(8.2%)	79(6.9%)	0.74
Number of diseased vessels2.8 ± 0.8		2.7 ± 0.6	0.37
**Intro-operation**			
Use of CPB	36(37.1%)	389(33.9%)	0.79
CPB time	110.8 ± 18.6	109.5 ± 15.8	0.45
ACC time	70.4 ± 13.7	68.2 ± 12.3	0.4
Bypass graftings	3.2 ± 0.8	3.1 ± 0.7	0.88
Operation time	187.4 ± 37.9	183.9 ± 40.7	0.55
**Post-operation(before re-intubation)**			
Relative hypoxemia	41(42.3%)	102(8.9%)	0.02
Pneumonia	23(23.7%)	68(5.9%)	0.02
Relative hypercapnia	17(17.5%)	57(5%)	0.08
LCOS	4(4.1%)	46(4%)	0.89
AMI	1(1%)	3(0.3%)	0.08
IABP	3(3.1)	68(5.9%)	0.32
AF	15(15.5%)	87(7.6%)	0.53
VF	1(1%)	2(0.1%)	0.08
AKI	8 (8.2%)	19(1.7%)	0.01
Stroke	2(2.1%)	25(2.2)	0.96
Re-operation for bleeding	5(5.1%)	52(4.5%)	0.57
Infection of incision	2(2.1%)	18(1.6)	0.47
TMVT (days)	3.8 ± 2.5	1.6 ± 0.5	0.01

BMI: body mass index; AMI: acute myocardial infarction; COPD: chronic obstructive pulmonary diseases; LVEF: left ventricular ejection fraction; LVEDD: left ventricular end-diastolic dimension; CHF: chronic heart failure; CPB: cardiopulmonary bypass; ACC: aortic cross clamping; LCOS: low cardiac output syndrome; IABP; intro-aortic balloon pump; AF: atrial fibrillation; VF: ventricular fibrillation; AKI: acute kidney injury; TMTV: total mechanical ventilation time.

Those variables with *p* <0.10 obtained through the univariate analysis were then entered into multivariate logistic regression analysis (Re-intubation or not as independent variable, variables with *p* <0.10 obtained through univariate analysis as dependent variables). As shown in Table 3, associated factors for re-intubation following CABG included preoperative COPD (OR = 2.134, 95%CI = 1.472-2.967), preoperative CHF (OR = 2.325, 95%CI = 1.512-3.121), postoperative relative hypoxemia (OR = 2.743, 95% CI = 1.657-3.326), postoperative AKI (OR = 2.976, 95% CI = 2.127-4.023), postoperative total mechanical ventilation time (OR = 1.976, 95% CI = 1.347-2.645). The Hosmer-Lemeshow goodness of fit coefficient of this model was 0.875.

**Table 3:** Factors for re-intubation through multivariate logistic regression analysis

**Factors**	**OR**	**95% CI**	**P**
Preoperative COPD	2.13	1.472-2.967	0.02
Preoperative CHF	2.33	1.512-3.121	0.01
Postoperative relative hypoxemia	2.74	1.657-3.326	0.01
Postoperative AKI	2.98	2.127-4.023	0.01
TMVT	1.9	1.347-2.645	0.02

The Hosmer-Lemeshow goodness of fit coefficient of this model was 0.875. OR: odds ratio; CI: confidence interval: COPD: chronic obstructive pulmonary diseases; CHF: chronic heart failure; AKI: acute kidney injury; TMTV: total mechanical ventilation time.

## Discussion

Improvement in cardiopulmonary performance, shorter ICU and hospital stay as well as reduction in costs could be achieved when cardiac surgical patients were weaned from mechanical ventilator at the appropriate time. Sometimes, appropriate prolonged ventilation support could have contributed to avoid endotracheal re-intubation. In this study, all the 1,244 patients were extubated within 48 hours after CABG. But among them, 97 patients were re-intubated after first extubated, with an incidence of 7.8%. The in-hospital mortality in the re-intubation group was more than six times that in the non-re-intubation group (9.3% versus 1.4%, *p* = 0.004). Besides the in-hospital mortality, re-intubation correlated with many negative outcomes such as pneumonia, tracheotomy, acute kidney injury, infection of incision, prolonged mechanical ventilation time, prolonged ICU stay and prolonged hospital stay. So, it became crucial for clinicians to evaluate the associated factors for endotracheal re-intubation following CABG. Through univariate analysis, the risk factors were as follows: age, BMI, preoperative COPD, preoperative CHF, preoperative LVEF, preoperative PaO2, postoperative relative hypoxemia, postoperative pneumonia, postoperative AKI and time of mechanical ventilation before re-intubation. Furthermore, through logistic regression analysis, the associated factors of re-intubation following CABG were as follows: preoperative COPD, preoperative CHF, postoperative relative hypoxemia, postoperative AKI and postoperative total mechanical ventilation time. The Hosmer-Lemeshow goodness of fit of the statistical model established in this study was 0.875, indicating that the model matches the data very well, therefore the results are statistically very reliable.

Postoperative acute kidney injury was an important associated factor for re-intubation following CABG. The mechanism of AKI after CABG is a complex interplay of many factors such as low renal perfusion during cardiopulmonary bypass, ischemic-reperfusion injury and systemic inflammatory reaction syndrome (SIRS). Some studies [3,4] indicated that SIRS and oxidative stress should be responsible for perioperative AKI in cardiac surgery. Postoperative AKI was an important adverse event, and was associated with many complications such as acute renal failure, chronic dialysis, prolonged ICU stay and hospital stay, higher cost and increased mortality [5,6]. The AKI and coexisted water-sodium retention can lead to pulmonary edema and CHF. If the water cannot be removed timely the pulmonary complication and re-intubation occurred inevitably. So patients suffering from postoperative AKI were prone to postoperative pulmonary complication even re-intubation. Thus proper management of postoperative AKI following CABG may contribute to reduce the incidence of re-intubation. A stable hemodynamic condition is the key requirement for the therapy of AKI. As a means of decreasing the risk of AKI after cardiac surgery, statin therapy has gained more and more attention [4]. Some drugs such as furosemide, low-dose dopamine (2-5 μg∙kg^-1^ min^-1^), natriuretic peptide, etc., have shown potential benefit in reducing the severity of renal injury as well as volume overload. Strengthened diuretic therapy and renal replacement are two effective methods for postoperative AKI. More and more evidence demonstrates that continuous renal replacement therapy is beneficial to the therapy of AKI. That beginning continuous renal replacement therapy early is extremely important [7].

Postoperative relative hypoxemia seem to be also an associated factor for re-intubation following CABG. Among the patients of post hypoxemia, five to fifteen percents were re-intubated [8]. Postoperative hypoxemia may cause rapid heartbeat and accelerated breathing. Lactate accumulation and inadequate tissue perfusion can decrease myocardial function and make homodynamic unstable. The extent of cardiopulmonary function damage is minor in relative hypoxemia. But if this low-level hypoxemia is not improved it may progress to serious hypoxemia and re-intubation following the first extubation. The reasons for postoperative relative hypoxemia most commonly included atelectasis and pulmonary edema. Pulmonary volume alteration can also lead to the postoperative hypoxemia. Using computed tomography, Rodrigues RR, *et al.*[9] investigated postoperative pulmonary alterations and their impact on blood oxygenation. Compared to preoperative CT, there was a 31% postoperative reduction in pulmonary gas volume while tissue volume increased by 19%. Non-aerated lung increased by 253 ± 97 g, from 3 to 27%, after surgery and poorly aerated lung by 72 ± 68 g, from 24 to 27%, while normally aerated lung was reduced by 147 ± 119 g, from 72 to 46%. So, effective measures should be taken to improve the lung oxygenation. Prompt prolonging mechanical ventilation and non-invasive positive pressure ventilation (NIV) after extubation can avoid the incidence of re-intubation effectively. NIV is safe and effective after cardiac surgery [10]. It may be beneficial to restore lung function more quickly, safely and well accepted by patients [11]. The vital capacity and lung volume decrease in obesity and its oxygen consumption increases. The influence of small amount of hydrothorax to obesity may he greater than that to normal body form. So, even if the hydrothorax is not too much in obesity it should be treated actively.

Preoperative congestive heart failure was also an important associated factor for re-intubation following CABG. Patients suffering from preoperative CHF often had pulmonary edema which in turn could have resulted in a change of pulmonary ventilation/blood flow ratio, thus resulting in postoperative hypoxemia. So, patients suffering from preoperative CHF were prone to postoperative hypoxemia even acute respiratory distress syndrome [12]. In addition, long-term respiratory muscle overwork may result in postoperative respiratory failure [13]. All of these may lead to postoperative prolonged mechanical ventilation so far as re-intubation. Thus, proper preoperative management of might contribute to reduce the incidence of re-intubation and ventilation time. Besides of positive inotropic drugs and diuretic, the natriuretic peptides gained more and more attention in the treatment of CHF during CABG. Sezai A, *et al.*[14] considered that in patients with left ventricular dysfunction undergoing CABG, human atrial natriuretic peptide (hANP) showed renal- and cardio-protective effects and reduced postoperative complications. Mentzer, *et al.*[15]. led the NAPA trial in which 272 patients with underlying left ventricular dysfunction CABG using CPB were randomized to nesiritide or placebo. Significant primary outcomes were peak increase in serum creatinine, a smaller decrease in glomerular filtration rate (GFR), a significant increase in urine output in 24 h postoperative period, a shorter ICU stay and reduced early mortality with nesiritide compared to placebo.

Preoperative severe COPD was one of the key associated factors of re-intubation following CABG. In our study, thirteen patients with a history of severe COPD were re-intubated due to postoperative carbon dioxide accumulation. Small airway obstruction and larger physiological dead space were observed in patients suffering from COPD. Furthermore, the surgery of CABG with cardiopulmonary bypass may reduce the volumes and capacities of lung. In addition, angina can lead to pulmonary changes together with COPD. Gimenes C *et al.*[16] observed that severe angina (class III), in association with COPD, results in higher reductions in pulmonary pressures between the preoperative period and the 5th postoperative day. The above-mentioned reasons can cause obstructive and restrictive ventilation disorder, and decrease vital capacity and maximal ventilation volume. So, postoperative re-intubation could have been possible.

The most commonly cause of re-intubation after CABG was pulmonary edema due to CHF. Secondly, hypoxemia due to pulmonary disease was also an important cause of re-intubation. Actually, hypoxemia due to cardiogenic and noncardiogenic disease was the main cause of re-intubation, which accounted for more than seventy percents. So, hypoxemia before and after extubation should be managed carefully. If the PaO_2_ before extubation is lower than 90 mmHg, delaying extubation may be a better choice. Extubation should not be carried out until the pulmonary edema and oxygenation are improved. If the PaO_2_ after extubation is lower than 90 mmHg, non-invasive positive pressure ventilation may be contributed to improve the oxygenation.

The retrospective nature and small sample size were the main limitation of this study. The relatively long time period this study covers may carry along variable changes in operative and postoperative practices that might have affected outcomes. The pulmonary function measured preoperatively may be influenced due to the mixing of cardiac dysfunction.

## Conclusions

Preoperative COPD, preoperative CHF, postoperative relative hypoxemia, postoperative AKI and postoperative total mechanical ventilation time were five independent risk factors for re-intubation following CABG. Re-intubation may be avoided by proper perioperative management of cardiopulmonary function. Decreasing of AKI may contribute to the prevention of re-intubation.

## Consent

Written informed consent was obtained from the patient for the publication of this report and any accompanying images.

## Abbreviations

ACC: Aortic cross clamping; AF: Atrial fibrillation; AKI: Acute kidney injury; AMI: Acute cardiac infarction; BMI: Body mass index; CABG: Coronary artery bypass graftings; CAD: Coronary artery disease; CHF: Congestive heart failure; COPD: Chronic obstructive pulmonary disease; CPB: Cardiopulmonary bypass; FEV1: Forced expiratory volume in 1 second; FVC: Forced vital capacity; IABP: Intra-aortic balloon pump; ICU: Intensive care unit; LCOS: Low cardiac output syndrome; LVEDD: Left ventricular end-diastolic diameter; LVEF: Left ventricular ejection fractions; TMVT: Total mechanical ventilation time; VF: Ventricular fibrillation.

## Competing interests

The authors declare that they have no competing interests.

## Authors’ contributions

LJ conceived of the study, and participated in its design and coordination and drafted the manuscript. SS participated in the statistical analysis. YM is in charge of English revision. YZ carried out the final version approval. All authors read and approved the final manuscript.
